# Promotion of prostatic metastatic migration towards human bone marrow stoma by Omega 6 and its inhibition by Omega 3 PUFAs

**DOI:** 10.1038/sj.bjc.6603030

**Published:** 2006-03-07

**Authors:** M D Brown, C A Hart, E Gazi, S Bagley, N W Clarke

**Affiliations:** 1 ProMPT Genito Urinary Cancer Research Group, Cancer Research UK Paterson Institute, Christie Hospital NHS Trust, Wilmslow Road, Manchester M20 4BX, UK; 2 Advanced Imaging Facility, Cancer Research UK Paterson Institute, Christie Hospital NHS Trust, Wilmslow Road, Manchester M20 4BX, UK; 3 Department of Urology, Christie Hospital NHS Trust, Wilmslow Road, Manchester M20 4BX, UK; 4 Department of Urology, Salford Royal Hospital NHST, Eccles Old Road, Salford, M6 8HD, UK

**Keywords:** prostate cancer, arachidonic acid, metastasis, bone marrow, Omega 6, Omega 3

## Abstract

Epidemiological studies have shown not only a relationship between the intake of dietary lipids and an increased risk of developing metastatic prostate cancer, but also the type of lipid intake that influences the risk of metastatic prostate cancer. The Omega-6 poly-unsaturated fatty acid, Arachidonic acid, has been shown to enhance the proliferation of malignant prostate epithelial cells and increase the risk of advanced prostate cancer. However, its role in potentiating the migration of cancer cells is unknown. Here we show that arachidonic acid at concentrations ⩽5 *μ*M is a potent stimulator of malignant epithelial cellular invasion, which is able to restore invasion toward hydrocortisone-deprived adipocyte-free human bone marrow stroma completely. This observed invasion is mediated by the arachidonic acid metabolite prostaglandin E_2_ and is inhibited by the Omega-3 poly-unsaturated fatty acids eicosapentaenoic acid and docosahexaenoic acid at a ratio of 1 : 2 Omega-3 : Omega-6, and by the COX-2 inhibitor NS-398. These results identify a mechanism by which arachidonic acid may potentiate the risk of metastatic migration and secondary implantation *in vivo*, a risk which can be reduced with the uptake of Omega-3 poly-unsaturated fatty acids.

Prostate cancer (CaP) is the second most common malignancy prevalent in men worldwide, comprising 11.9% of all cancer cases diagnosed in 2002. There is considerable geographic variation in incidence, the disease being more common in the developed countries (19%) than in developing countries (5.3%) (Parkin *et al*, 2005). Even within the developed countries there is considerable variation in the rate of presentation, with the USA having the highest age-standardised incidence (124.8/100 000) and Japan the lowest (12.6/100 000). This is also reflected in the mortality rates, whereby North America, Northern and Western Europe have high age-standardised rates (15.8, 17.5 and 19.7/100 000, respectively) as compared to Japan (5.7/100 000). Migrants from low- to high-risk countries show a marked increase in risk, reaching levels of cancer risk similar to that in resident populations (Haenszel, 1961; Haenszel and Kurihara, 1968). Although the aetiology and pathogenesis of CaP is unknown, various epidemiological studies have shown a relationship between dietary lipids and development of the disease (Snowdon *et al*, 1984; Giovannucci *et al*, 1993). Also there is increasing evidence that it is not only the quantity of lipid but the type of lipid intake that influences the risk of CaP. Omega-6 poly-unsaturated fatty acids (*ω*-6 PUFA) have been shown to promote CaP, whereas Omega 3 (*ω*-3) PUFA, from marine foods, may inhibit oncogenesis (Norrish *et al*, 1999).

Prostate cancer has a predilection to metastasise to the bone marrow stroma (BMS) of the axial skeleton. Men who develop CaP bone metastases will almost invariably die from their disease in the absence of an intercurrent illness, the median time between clinical presentation with bone metastases and death being 18 months (George, 1988). The mechanics of invasion through the endothelium into the BMS is a complex and multi-step process, which is only just beginning to be understood with the utilisation of *in vitro* invasion chambers. These *in vitro* models allow not only the study of the mechanism of endothelial transmigration, but also the determination of specific BMS attractants. The exact nature of the stimulatory mechanisms which direct the PEC to the BMS are currently unknown although some components have been identified. The CXCR4/SDF-1 signalling pathway is a potent stimulator of PEC invasion (Taichman *et al*, 2002; Sun *et al*, 2003). However, in comparison to human BMS, it forms only a small part of the overall stimulus (Hart *et al*, 2005). The BMS is a lipid-laden environment, rich in various lipids including the *ω*-6 PUFA's, linoleic acid (LA) (4.1±0.84% to 15.3±2.9%) and arachidonic acid (AA) (2.5±0.9 to 9.52±0.4%) (Sumida, 1965; Denizot *et al*, 1998; Denizot *et al*, 1999).

Studies of prostate epithelia have demonstrated the importance of lipid/cellular interactions. Mamalakis *et al* (2002) showed that relative to benign disease patients, the adipose tissue and prostatic tissue of CaP patients had reduced levels of AA and the *ω*-3 lipid docosahexaenoic acid (DHA), and a reduced *ω*-3 : *ω*-6 PUFA ratio. Adipocyte/epithelial cell interactions have been shown to induce proliferation of prostate (Tokuda *et al*, 2003) and mammary (Rahimi *et al*, 1994) cancer cell lines and plays an important role in normal breast development (Zangani *et al*, 1999).

Omega-6 PUFA's, especially the LA metabolite AA, has been associated with prostate cancer progression (Honn *et al*, 1994; Norrish *et al*, 1999), stimulating proliferation and inhibiting apoptosis (Ghosh, 2004). These effects are mediated by the increase in the cyclooxygenase-2 (COX-2) product prostaglandin E_2_ (PGE2) (Hughes-Fulford *et al*, 2005), and lipoxygenase (LOX) products 5 and 12(S)-HETE (hydroxyeicosatetraenoic acid). 5-hydroxyeicosatetraenoic acid has been shown to induce PC-3 proliferation (O'Flaherty *et al*, 2002) and loss of 5-LOX expression in both DU145 and PC-3, by activation of the orphan nuclear receptor ROR*α*, has been shown to inhibit the proliferative effects of both AA and LA (Moretti *et al*, 2004). 12(S)-hydroxyeicosatetraenoic acid, does not induce PC-3 proliferation but induces endothelial invasion (angiogenesis) leading to increased proliferation *in vivo* (Nie *et al*, 1998).

The stimulatory ability of AA can be blocked by the addition of the *ω*-3 PUFAs, eicosapentaenoic acid (EPA) and DHA. This may be due to *ω*-3 PUFA modifying the AA biosynthetically derived prostaglandins and eicosanoids via competitive inhibition of the COX and LOX pathways (Karmali *et al*, 1987). Rose (1997) showed using malignant breast and PEC that both EPA and DHA not only blocked AA synthesis from LA by competition for Δ4 desaturase but also blocked prostaglandin and HETE synthesis from AA by direct competition for COX and LOX enzymes.

Epidemiological data suggest that the dietary ratio of *ω*-3 : *ω*-6 PUFA is crucial in determining the risk of metastatic CaP. Here we present for the first time *in vitro* data showing that AA is a key attractant of metastatic PECs to human BMS and that this stimulus can be blocked in a PGE2-dependent manner by the addition of *ω*-3 PUFAs.

## MATERIALS AND METHODS

### Materials

All Reagents were purchased from Sigma-Aldridge (Poole, UK) except AA, 5-HETE and 12(S)-HETE, which were supplied by MP Biochemicals UK (London, UK). All fatty acids were made up to 10 mg ml^−1^ in ethanol. All tissue culture medium and horse serum was from Invitrogen (Paisley, UK) with the exception of foetal calf serum (FCS) supplied by Labtech International Ltd (Uckfield, East Sussex, UK). Tissue culture plastic, Matrigel® Basement Membrane Matrix and 8 *μ*m cell culture inserts were from Becton Dickinson Labware (NJ, USA). Iwaki quartz-based 35 mm Petri dishes were supplied by Bibby Sterilin (Staffordshire, UK). Oil Red O was obtained from VWR International Ltd (Leicestershire, UK). The inhibitors NS-398 was from Alexis Corp and MirrIR slides from Keveley Technologies (Ohio, USA).

### Antibodies

Mouse anti-human pan cytokeratin, Nile Red and DAB tablets were from Sigma-Aldridge (Poole, UK); rabbit anti mouse biotinylated antibody from DAKO Ltd (Cambridge, UK) and Vectastain Elite ABC kit from Vector Laboratories (CA, USA).

### Cell culture

The PC-3 cell line (Kaighn *et al*, 1979) was cultured in Ham's F12 (Sigma) and 7% FCS and grown at 37°C in a humidified atmosphere of 5% CO_2_ in air. Bone marrow stroma was cultured from human ribs removed for access during routine renal surgery after informed consent (ProMPT LREC 02/ST/122) and prepared for tissue culture using the method of (Coutinho *et al*, 1993). The cultures were grown at 33°C in 5% CO_2_ in air for 4–5 weeks until haemopoietically active areas were observed.

### Co-culture experiments

Bone marrow stroma cultures were trypsinised and re-seeded into Iwaki quartz-based 35 mm Petri dishes at the same cell density and left to re-establish for 14 days. Bone marrow stroma media was replaced with medium containing 500 PC-3 cells/dish. The co-cultures were incubated for 48 h at 37°C then fixed in 4% paraformaldehyde.

### Immunocytochemistry

After fixation co-cultures were permeabilsed using ice-cold methanol, blocked with 10% rabbit serum followed by 0.3% hydrogen peroxide. Co-cultures were incubated with mouse anti-human pan cytokeratin at 1 : 200 followed by biotinylated rabbit anti-mouse 1 : 400. A complex of avidin DH and biotinylated horseradish peroxidase H was then added and developed with DAB substrate. Adipocytes were stained by Oil Red O. Briefly, cultures were rinsed in 60% isopropanol then incubated in a 0.5% Oil Red O in 60% isopropanol solution for 10 min. Cultures were differentiated for a few minutes in fresh 60% isopropanol then rinsed with water.

### Brightfield volumetric analysis

PC-3 and adipoctye cells stained with Oil Red O were analysed in colour. Planes of focus were visualised utilising a Zeiss AxioVert 35 M with a C-Apochromat × 63 1.2NA water immersion objective lens under brightfield illumination. A stepper motor (Ludl electronics, NY, USA) was attached which permits fine control of the focus (0.2 micron steps). Images were then accumulated into a stack utilising ImageJ Image Analysis software (Rasband, 2005). A depth of focus algorithm was applied to each colour component of the volume, in greyscale, and the volume subsequently merged to form a colour volume of data. The image was then viewed in Imaris (Bitplane) and a cross sectional view of the data generated.

### Fourier transform infrared microscopy

Prostate bone metastases paraffin-embedded sections, isolated from consenting patients, were mounted on MirrIR slides and deparaffinised with Citroclear followed by 20 min acetone treatment before air drying. High-definition FTIR microspectroscopic maps of 6.25 *μ*m pixel resolution of bone marrow tissue was collected in rapid-scan mode using a Perkin Elmer Spotlight spectrometer and a 16 × 1 MCT linear array detector. Mid-IR spectra within the wavenumber range 4000–748 cm^−1^ were collected in reflection mode. The background scan was recorded at 8 cm^−1^ spectral resolution with 75 scans, whereas the sample scan was recorded at 8 cm^−1^ spectral resolution with 60 scans. Fourier transform infrared microscopy spectral images were processed with Spotlight version 1.0.1.

### Invasion assay

PC-3 invasion was assessed using the method described by Hart *et al* (2005). Briefly, Matrigel-coated cell culture inserts were placed in a 24-well plate containing 1 ml of DMEM/0.1% fatty acid-free BSA with either plain tissue culture plastic (TCP-negative control), BMS (positive control) or escalating concentrations of AA, DHA, EPA, 5-HETE, 12(S)-HETE, 15(S)-HETE and Prostaglandin E_2_ (PGE2). The COX-2 inhibitor NS-398 was added at 8 *μ*M. PC-3 cells, serum starved for 24 h in RPMI 1640 medium, were seeded at 1 × 10^5^ per insert then incubated at 37°C for 18 h after which inserts were fixed and stained in 2% crystal violet and counted according to manufacturer's instructions using a grid graticule.

### Uptake of AA in PC-3 cells using flow cytometry

PC-3 cells were serum starved for 24 h in RPMI 1640 medium, trypsinised and placed in RPMI serum-free medium plus or minus AA at 10 *μ*M at a concentration of 2 × 10^5^ cells/ml. The cells were incubated in these conditions at 37°C in 5% CO_2_ in air for up to 180 min before fixing and staining with 5 *μ*M Nile Red for 5 min. Analysis was carried out using a FACS Vantage SE equipped with an argon ion laser running at 200 mW 488 nm. Emission in the 560–565 nm range yellow/gold was measured using linear amplification. All cells were analysed within 30 min.

### AA Uptake by PC-3 cells visualized using spinning disc confocal microscopy

PC-3 cells were plated onto quartz-bottomed 35 mm petri dishes and grown to semi confluence. Cells were then serum starved in RPMI 1640 medium for 24 h before use. Before visualisation on the microscope, the media was exchanged for RPMI serum-free media (without phenol red) and 5 *μ*M Nile Red. Cells were visualised on a PerkinElmer Ultraview situated on a Zeiss Axiovert 200 M with a full environmental chamber and heated objective set at 37°C. Nile Red was excited using the 488 nM laser and the cells photographed every 5 min for 2 h in the *z*-axis and combined to give a continuous sequence.

### Statistics

All values are presented as mean±s.e.m. All assays were compared by use of the two-tailed Student's *t*-test. A threshold of significance was set at *P*<0.05.

## RESULTS

### Localisation of malignant PEC to BMS adipocytes and lipid uptake

**Figure 1:**
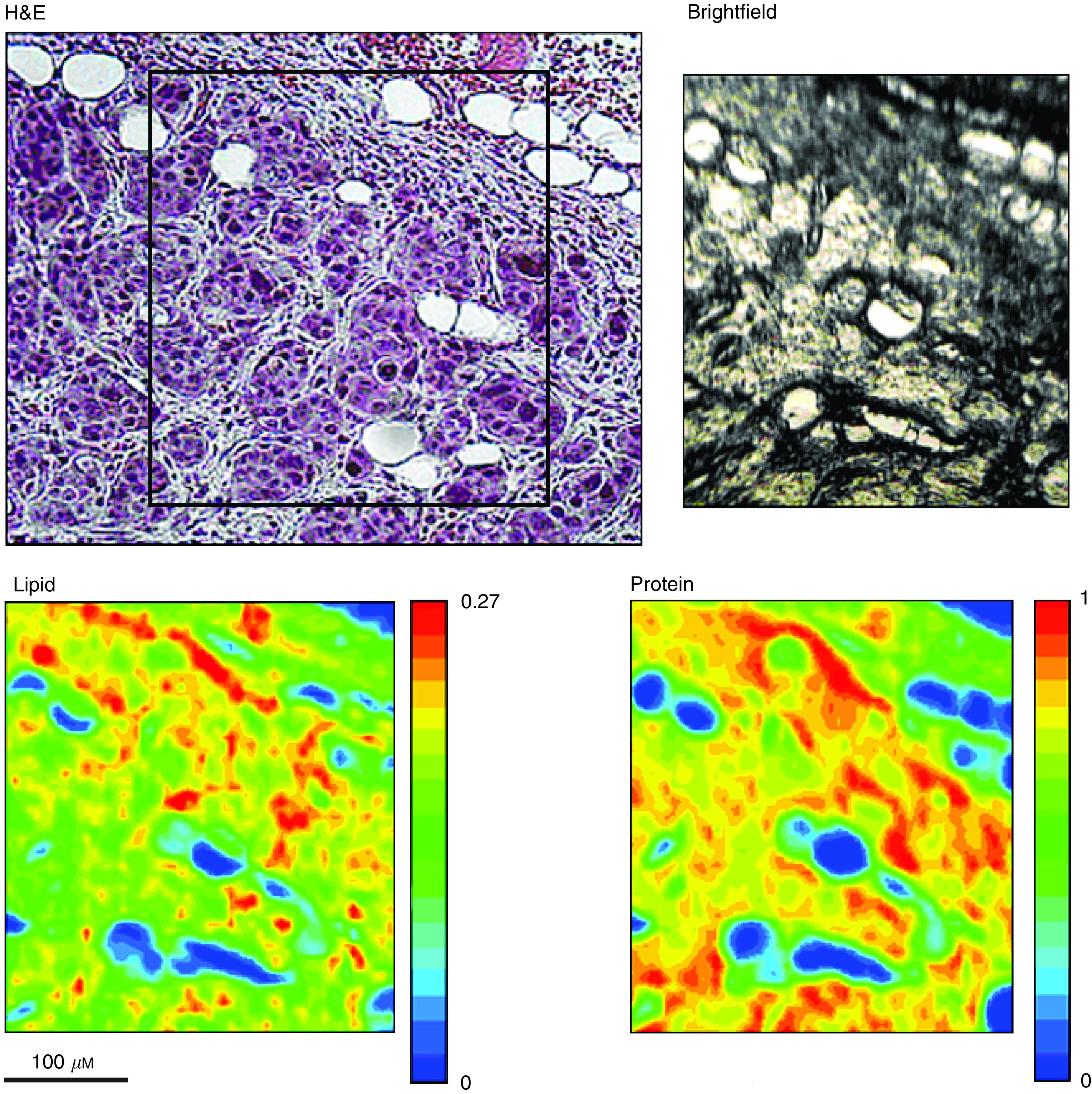
PECs localise to lipid-rich regions in bone marrow metastases. Fourier transform infrared microscopy chemometric analysis of prostate bone metastasis. Haematoxylin and eosin-stained section (H&E) depicting bone metastasis. Square outlines the region of a serial section analysed by FTIR as shown by the brightfield image. The lighter shaded locations within the brightfield photomicrograph depict CaP cells. The lipid hydrocarbon peak area (3007 cm^−1^ to 2798 cm^−1^) intensity image displays lipid localisation within the section. The protein image (1729 cm^−1^ to 1485 cm^−1^) reveals the boundary between the CaP cells and haemopoietic bone marrow. Scale bar=100 *μ*m.

Tokuda *et al* (2003) showed that human PECs interact with adipocytes *in vitro* with a resultant increase in proliferation and differentiation of the PECs. We therefore analysed human prostate bone metastases chemometrically using Fourier transform infrared microscopy (FTIR), to determine whether PECs associate with lipid-rich regions within the BMS *in vivo*. Figure 1 shows serial sections of a human prostate bone metastasis stained with haematoxylin and eosin or examined chemometrically by FTIR. The spectral maps show the localisations of protein and lipid signals within the tissue, with blue representing the lowest signal and red the highest. The intensity distribution of the lipid hydrocarbon signal (using the ν-band region between 3007 and 2978 cm^−1^) demonstrates a strong lipid signal associated with/around the prostate bone metastasis suggesting either lipid uptake by the PECs or an association with lipid-rich regions within the BMS. It has also been observed in our previous studies of PECs co-cultured in human long-term bone marrow cultures (Lang *et al*, 1997; Lang *et al*, 1998; Scott *et al*, 2000; Hart *et al*, 2002; Hart *et al*, 2005) malignant human PECs formed colonies in close proximity to adipocytes (unpublished finding).

**Figure 2:**
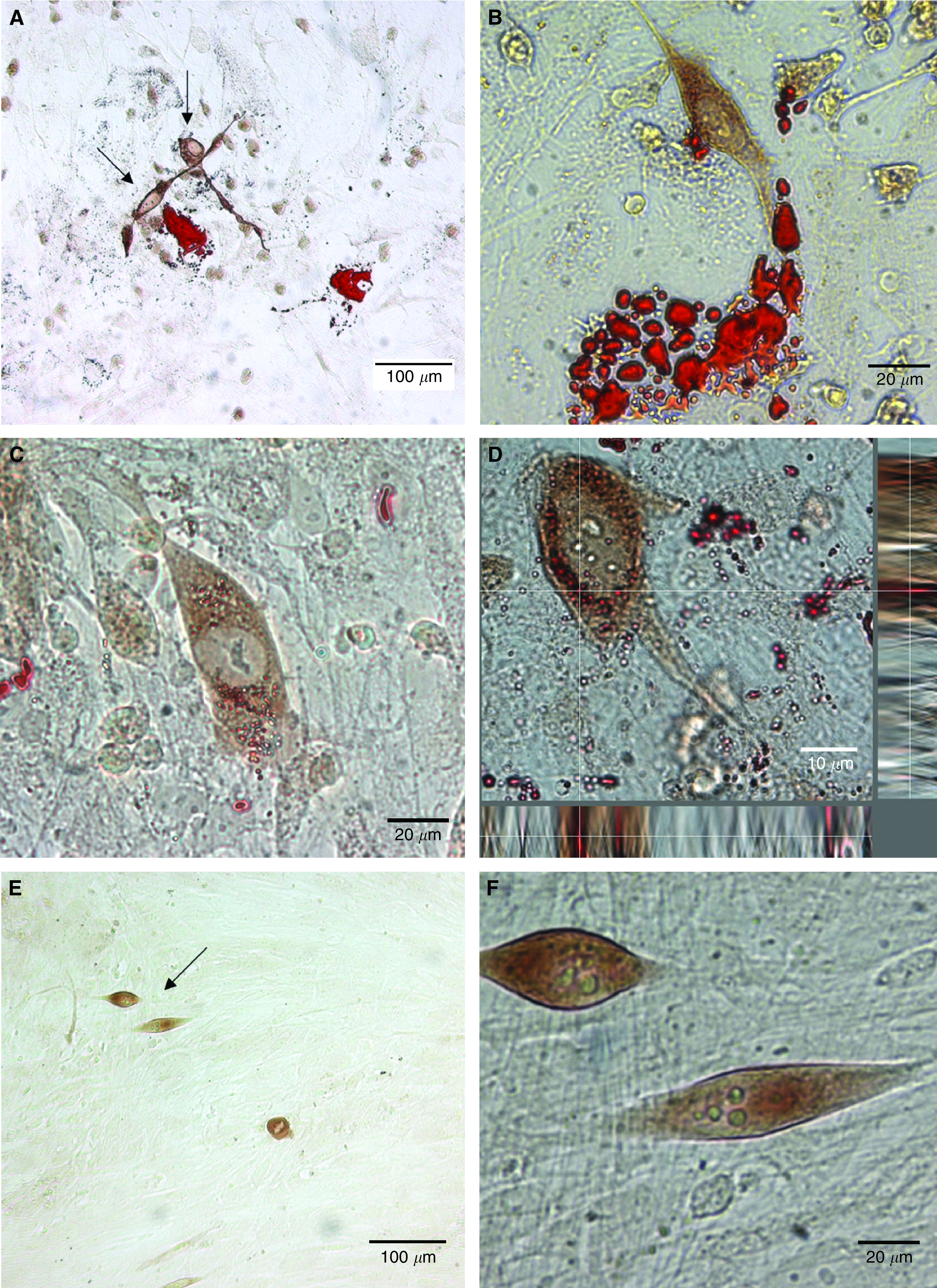
PC-3 cells localise to adipocytes in BMS co-culture and take up lipids. 5 × 10^2^ PC-3 cells co-cultured with primary long-term human BMS, visualised by staining with anti pan-cytokeratin and developed by DAB (brown). Bone marrow adipocytes and lipid droplets were visualised by staining with 0.5% Oil Red O. Images were captured utilising a Zeiss AxioVert 35 M with a C-Aprochromat × 63 1.2 NA water immersion objective lens. (**A**–**C**) Brightfield photomicrographs of PC-3 cells co-cultured with BMS showing PC-3 localisation to bone marrow adipocytes and lipid uptake. (**D**) Deconvolved brightfield image showing localisation of lipid droplets within the PC-3 cytoplasm. *x* and *y* panels show orthogonal planes of data along the white cross hair. (**E**–**F**) Brightfield photomicrograph of PC-3 cells co-cultured with primary human prostate fibroblasts.

We therefore hypothesised that malignant PECs may migrate towards adipocytes and utilise the lipids that they contained. To test this hypothesis, the PEC line PC-3 was seeded on to confluent long-term human BMS (Figure 2A–D) or primary prostate stroma (Figure 2E and F). PC-3 cells and lipid droplets were then visualised by staining with anti pan-cytokeratin, developed with DAB, and Oil Red O, respectively. Figure 2A and B illustrates that PC-3 cells migrate towards and form colonies around lipid-rich areas. High-resolution brightfield microscopy of PC-3 cells surrounding/in close proximity to lipid droplets shows that PC-3 cells take up lipids from the surrounding area of BMS (Figure 2C and D). This observation was confirmed using brightfield volumetric analysis of the PC-3/BMS co-culture. Figure 2D shows a de-convolved image of a PC-3 cell dual stained with anti pan-cytokeratin and Oil Red O with Z plane data collected at 0.2 *μ*M steps. Along both the *x* and *y* axis are orthogonal planes of data bisecting a lipid droplet, confirming its location within the PC-3 cell.

Within the prostate stroma, which lacks lipid centres, the PC-3 cells do not congregate, appear as isolated cells within the stroma and do not have Oil Red O-stained lipid droplets within the cytoplasm (Figure 2E and F).

### Uptake of AA by PC-3 cells *in vitro*

**Figure 3:**
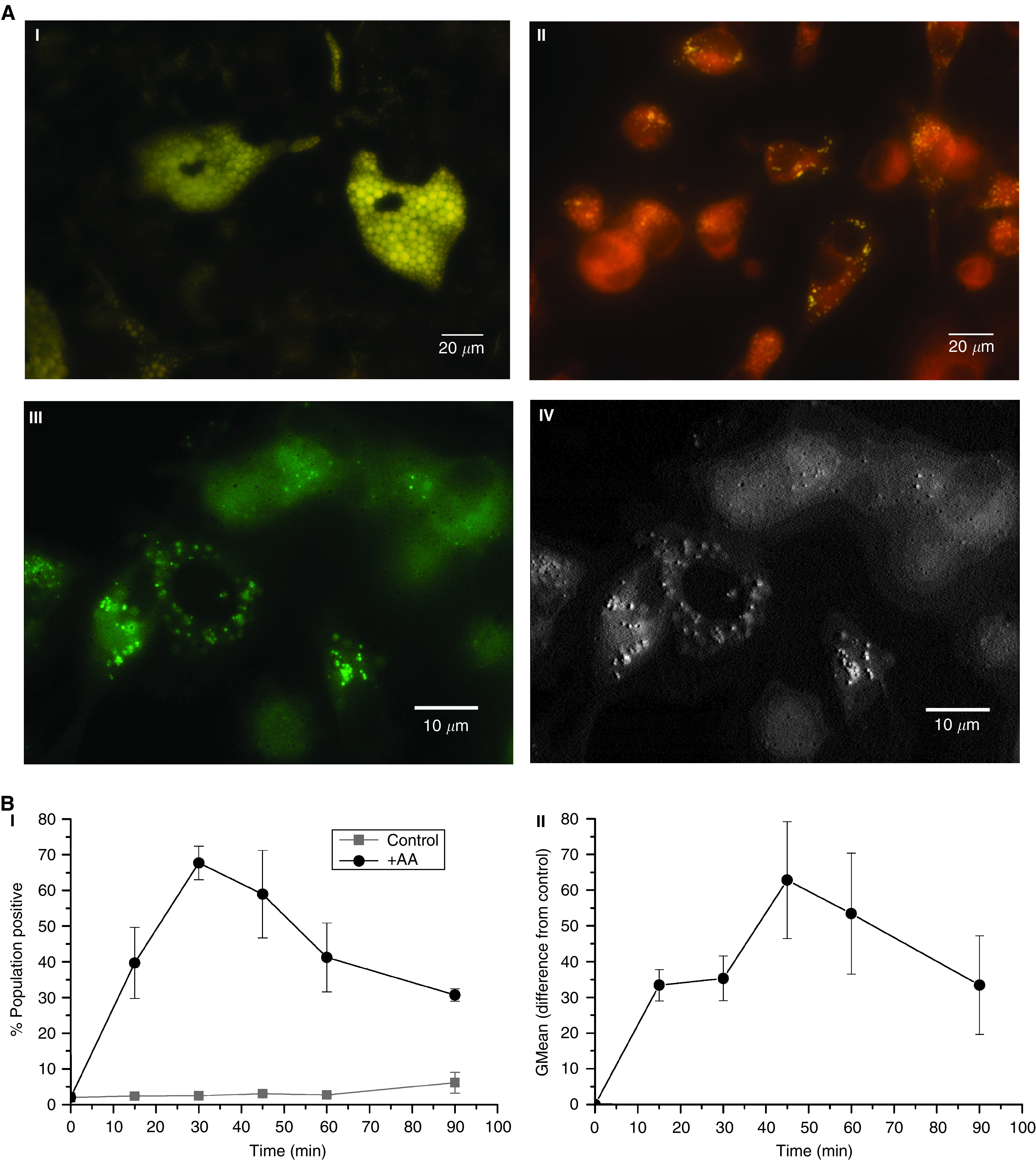
PC-3 cells rapidly take up the *ω*-6 PUFA AA from the microenvironment. (**A**) The lipid content of (I) bone marrow adipocytes or (II) PC-3 cell after loading with AA was visualised by fluorescent yellow/gold Nile Red staining (488 nm excitation–565 nm emission). (III) High-resolution confocal false coloured image of AA pulsed PC-3 cells. (IV) Confocal 3D relief image showing localisation of lipid droplets within the cytoplasm of PC-3 cells. (**B**) FACS analysis following the uptake of AA by serum-starved PC-3 cells over time after Nile Red staining; (I) graph depicting the percentage of the population staining positive for AA uptake overtime, (II) graph showing the level of AA uptake by PC-3 cells overtime as determined by the geometric fluorescent mean.

Hughes-Fulford *et al* (2005) showed that in the presence of an albumin carrier, uptake of AA by PC-3 cells could be detected after 2.5 h. To determine the rate of lipid uptake from their environment in the absence of carrier, we conducted a series of monoculture experiments with 10 *μ*M albumin-free AA. Figure 3A I and II show adipocytes and PC-3 cells in the presence of AA, stained with 5 *μ*M Nile Red and visualised by fluorescent microscopy. The adipocytes are densely packed with yellow-staining lipid droplets. These are also observed in the PC-3 cells, although at a much lower frequency. This observation was confirmed by confocal imaging (Figure 3A III), and the internalisation and localisation of the lipid droplets was verified by 3D confocal microscopy (Figure 3A IV). Utilisation of a fluorescent lipid stain enabled the uptake of AA by PC-3 cells to be monitored over time (Figure 3B). PC-3 cells were incubated with 10 *μ*M AA, fixed and stained with 5 *μ*M Nile Red at different time points. The number of cells and the amount of AA taken up by the PC-3 cells was determined by flow cytometry and followed in real time by spinning disc fluorescent microscopy. Arachidonic acid is rapidly taken up by PC-3 cells with a maximal number of cells, 67.71±4.72%, taking up AA at 30 min. After this time there is a steady decline in the number of AA positively stained cells, with only 30.73±1.73% of PC-3 cells staining positive 90 min post dosing. Maximum uptake occurs at 45 min, with a geometric mean difference to the controls of 62.83±16.39 (*P*=0.019), a figure which again declines with time. This phenomenon was also confirmed in real time with time lapse spinning disc microscopy (Supplementary Figure 1).

### Effect of *ω*-3 and *ω*-6 PUFAs on invasion

Omega-6 PUFAs, in particular AA, have been associated with an increase in risk of advanced CaP and that this risk can be reduced by increased intake of *ω*-3 PUFAs. Therefore, we sought to determine the ability of AA, DHA and EPA to stimulate PC-3 invasion through a synthetic basement membrane, Matrigel, and to compare its ability to stimulate migration with human BMS.

**Figure 4:**
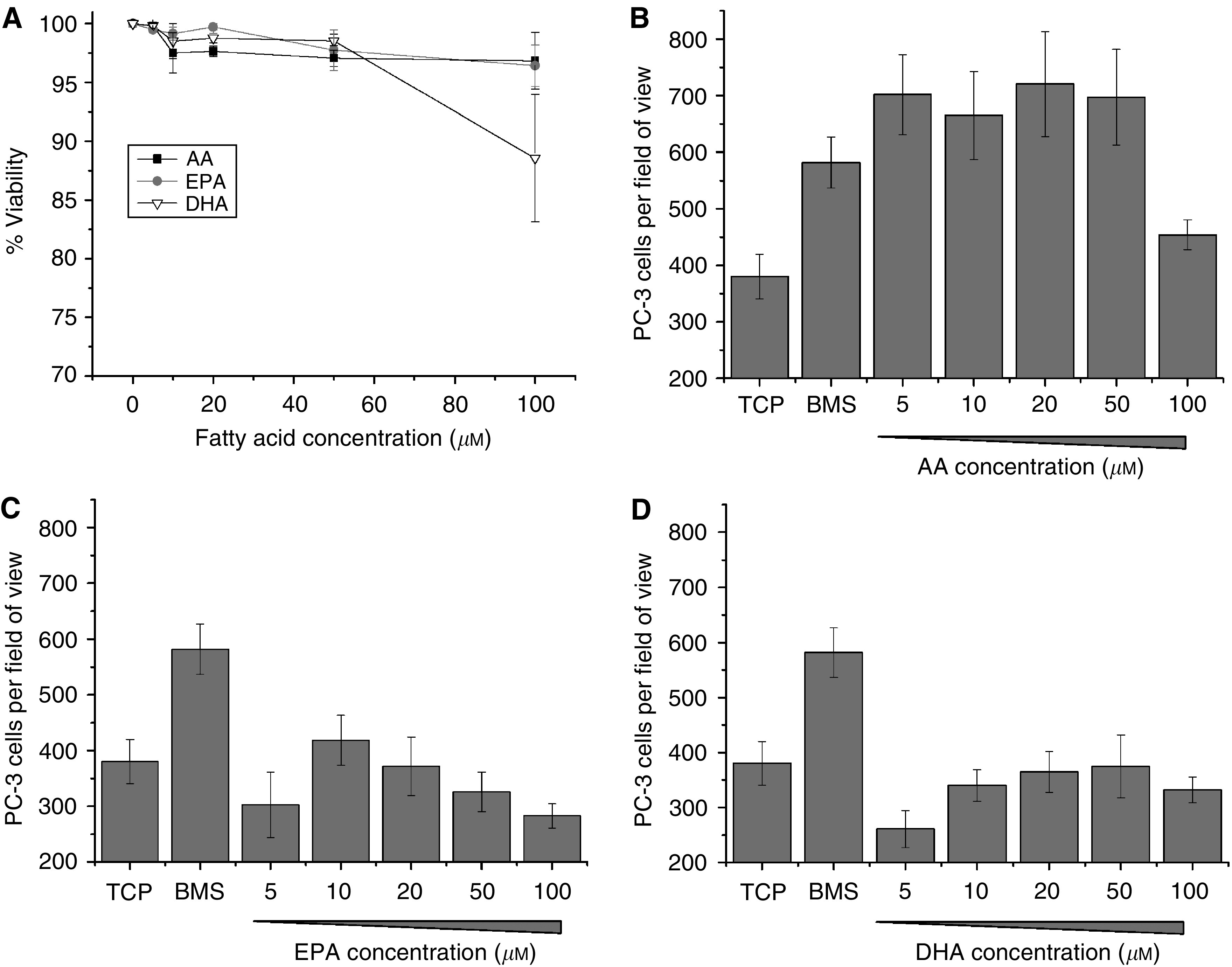
Omega-3 and *ω*-6 PUFAs do not reduce PC-3 cell viability but only AA induces cellular invasion. (**A**) Graph showing the effect of escalating doses of AA, DHA or EPA on PC-3 cell viability 18 h post dosing as determined by trypan blue exclusion (*n*=2). (**B**) Histograms showing the stimulatory effect of increasing concentrations of AA, (**C**) EPA and (**D**) DHA on the number of PC-3 invading through Matrigel over 18 h. Data represents mean number of cells per field of view plus standard error bars generated from four independent experiments (*n*=4).

PC-3 cells were cultured overnight in the presence of escalating doses of AA, DHA or EPA and cell viability assessed by Trypan blue exclusion. Only 100 *μ*M DHA was shown (Figure 4A) to have an effect on the overall cell viability of the PC-3 culture after 18 h exposure, reducing viability to 88.56±5.4% (although this did not reach statistical significance (*P*=0.168)).

To determine the ability of *ω*-3 and *ω*-6 PUFA to induce PEC invasion, exponentially growing PC-3 cells were seeded on to Matrigel-coated cell culture inserts above either TCP, human BMS or escalating doses (5–100 *μ*M) of AA, DHA or EPA. After 18 h at 37°C with 5% CO_2_, the number of invasive cells was counted. Arachidonic acid at concentrations ⩽50 *μ*M, was shown to be a potent stimulus for invasion (Figure 4B), with concentrations of ⩾5 *μ*M inducing similar levels of invasion to BMS (5 *μ*M AA; *P*=0.17). Arachidonic acid did not induce LnCaP or PNT2-C2 cell lines to invade even in the presence of androgen (Supplementary Figure 2).

In comparison, both EPA and DHA (Figure 4C and D) did not significantly induce invasion above the background level of TCP (*P*⩾0.05), except for 10 *μ*M EPA, which induced a small but nonsignificant increase in invasion of PC-3 cells (418.5±45.2 *vs* 380.3±39.6; *P*=0.347). Eicosapentaenoic acid concentrations ⩾50 *μ*M induced a reduction in the number of invading PC-3 cells in comparison to TCP (325.9±35.2 and 283±21.6 *vs* 380.3±39.6 at 50 *μ*M and 100 *μ*M, respectively) although these decreases were not significant (*P*=0.576 and *P*=0.144, respectively). With DHA at a 5 *μ*M concentration a nonsignificant reduction in cellular invasion was observed (260.8±33.8 *vs* 380.3±39.6; *P*=0.098) by comparison with TCP.

### *ω*-3 inhibits *ω*-6-stimulated PEC invasion

**Figure 5:**
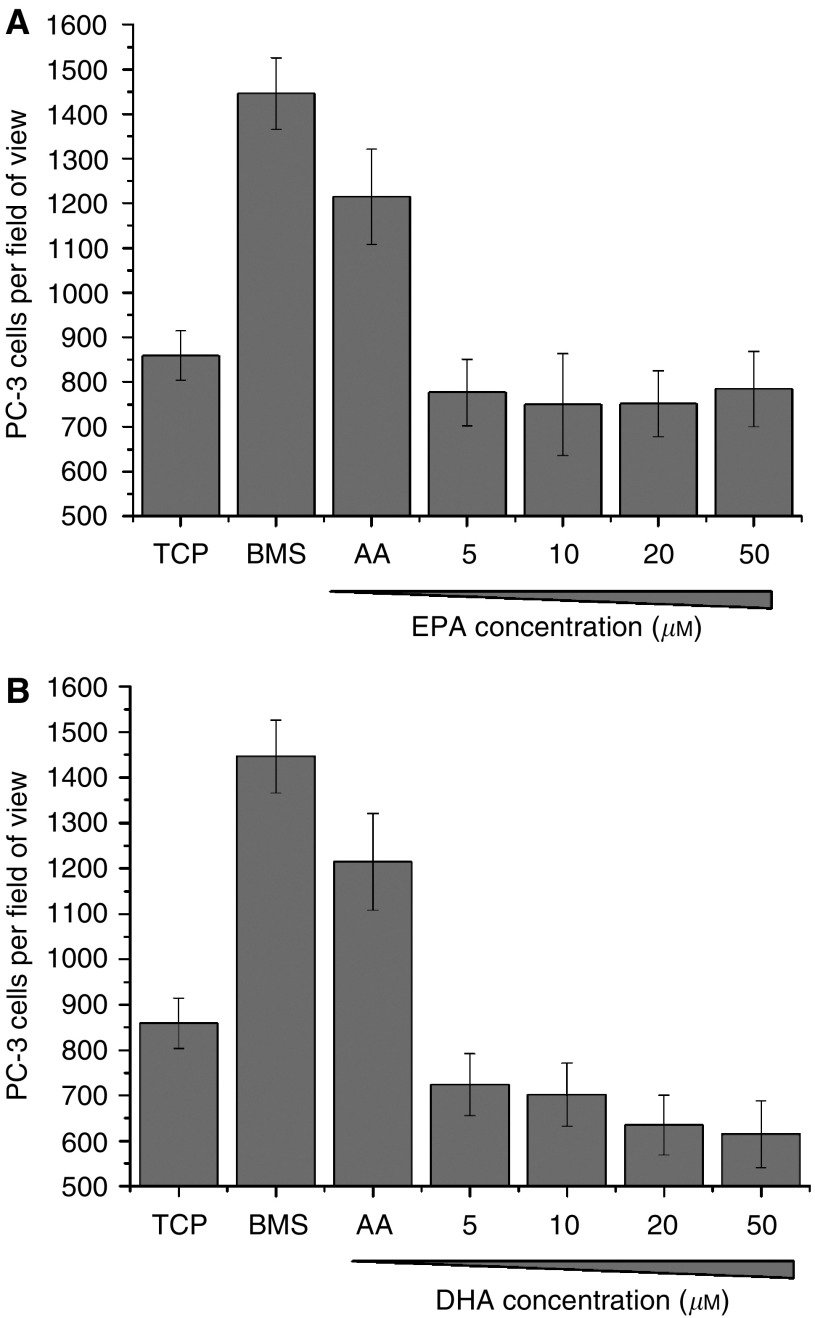
Addition of *ω*-3 PUFAs inhibits *ω*-6-induced PC-3 invasion. Histograms showing the effect of increasing concentrations of (**A**) EPA or (**B**) DHA on PC-3 invasion through Matrigel over 18 h towards 10 *μ*M AA. Data represents mean number of cells per field of view plus standard error bars generated from three independent experiments (*n*=3).

The proliferative potential of AA has been shown to be abrogated by the addition of *ω*-3 PUFA's (Honn *et al*, 1994; Rose, 1997; Norrish *et al*, 1999). We sought to determine whether the addition of *ω*-3 PUFAs to an environment rich in AA would reduce the level of induced invasion. Figures 5A and B shows the effect of the addition of increasing concentrations of EPA or DHA to an invasion chamber containing 10 *μ*M of AA as a stimulus and compared to TCP and BMS.

Both EPA and DHA were potent blockers of AA stimulation of invasion. Eicosapentaenoic acid and DHA, at concentrations ⩾5 *μ*M completely inhibited invasion towards 10 *μ*M AA, inducing similar levels of invasion as TCP (776.8±74.1 *vs* 858.7±55.1; *P*=0.396 and 723.7±68.1 *vs* 858.7±55.1; *P*=0.154 by comparison with TCP for 5 *μ*M EPA and DHA, respectively).

### PGE2 recovers *ω*-6 induced invasion in the presence of *ω-*3

To determine the basic mechanism of *ω*-3 inhibition of AA-induced malignant PEC invasion, we repeated the invasion assays with 10 *μ*M AA blocked with 20 *μ*M *ω*-3 PUFA and attempted recovery of invasion by the addition of the AA metabolites, 5-HETE, 12(S)-HETE, 15(S)-HETE and PGE2. All AA metabolites were titrated for their ability to stimulate invasion of PC-3 cells through a Matrigel basement membrane and used in subsequent experiments at the optimum concentration (data not shown).

**Figure 6:**
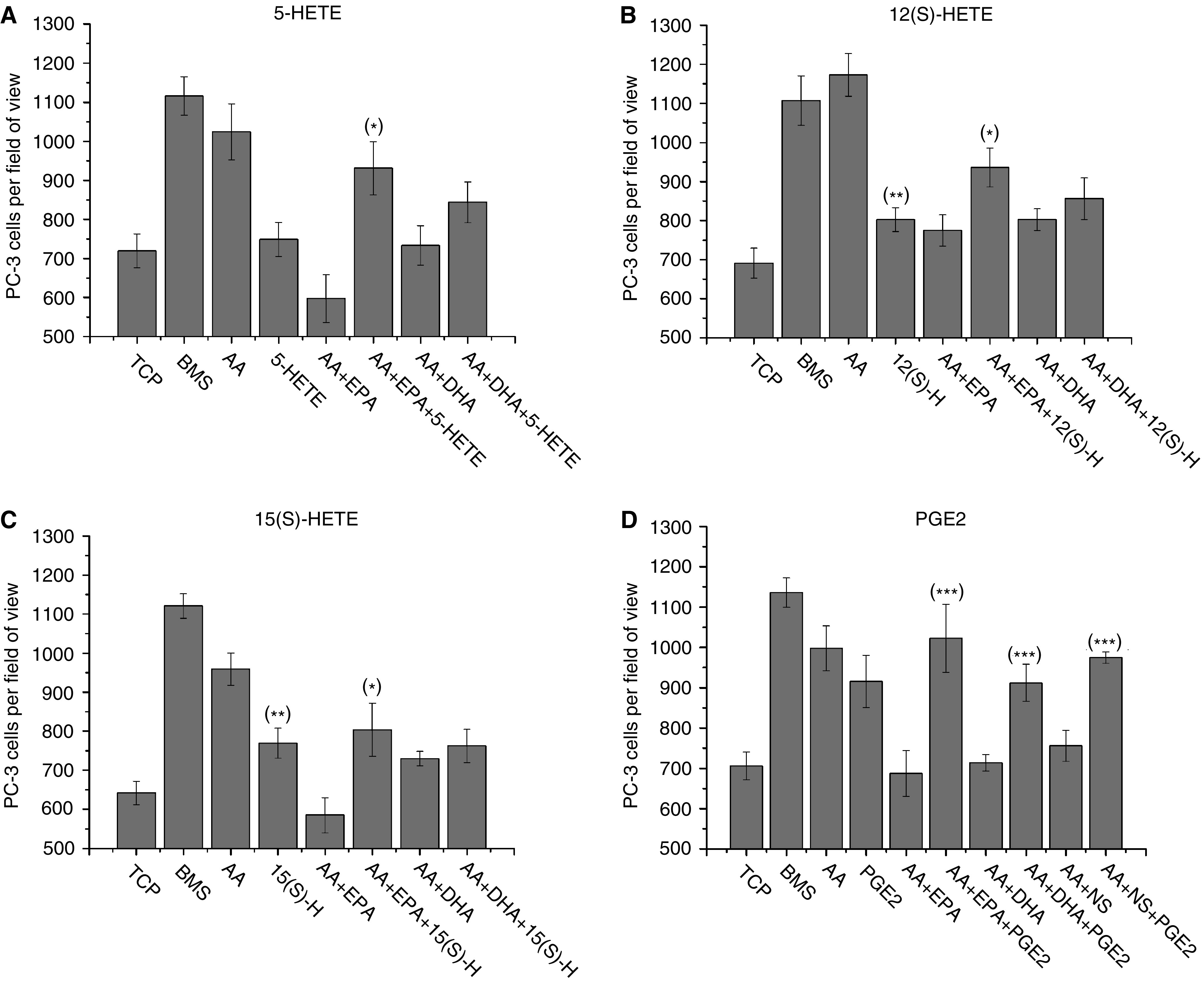
Inhibition of AA-induced invasion by DHA and EPA is via the blockade of prostaglandin E_2_ synthesis. Histograms showing the effect of the AA metabolites, 5-HETE (100 nM), 12(S)-HETE (300 nM), 15(S)-HETE (1 *μ*M) or PGE2 (10 ng/ml), on restoring PC-3 cell invasion through Matrigel towards AA (10 *μ*M) following blockade by the addition of either 20 *μ*M EPA, DHA or the COX-2 inhibitor NS398. Data represents mean number of cells per field of view plus standard error bars generated from six independent experiments (*n*=6). ^*^=*P* value ⩽0.05 as compared to *ω*-3 control; ^**^=*P* value ⩽0.05 as compared to TCP control; ^***^=*P* value ⩾0.05 as compared to AA.

Figure 6A–C show the effect of the addition of AA lipoxygenase products, 5-HETE, 12(S)-HETE and 15(S)-HETE on PC-3 invasion towards 10 *μ*M AA in the presence of blocking concentrations of DHA or EPA (20 *μ*M). Although 5-HETE (Figure 6A) did not induce invasion in its own right (748.7±43.3 *vs* 719.6±43.4 (5-HETE *vs* TCP), *P*=0.64), addition of 100 nM 5-HETE to AA blocked with 20 *μ*M EPA induced significant recovery of PC-3 invasion (931.3±68.4 *vs* 719.6±43.4; *P*=0.00457) as compared to EPA control. Although addition of 100 nM 5-HETE to AA blocked with 20 *μ*M DHA induced PC-3 invasion as compared to the DHA control, it was not significant (844±52.4 *vs* 719±43.4; *P*=0.1425).

15(S)-hydroxyeicosatetraenoic acid (Figure 6B) induced invasion of PC-3 cells as compared to TCP (769±38.7 *vs* 641.8±29.8; *P*=0.016) and partially restored PC-3 invasion in the presence of EPA-blocked AA (803.3±67.6 *vs* 584.5±44.6, *P*=0.0222 by comparison with the EPA blocked AA control). However, addition of 15(S)-HETE did not release the DHA blocked system (*P*=0.488**7** as compared to DHA control). 12(S)-hydroxyeicosatetraenoic acid like 15(S)-HETE (Figure 6C) induced invasion of PC-3 by itself, inducing 802.6±30.4 *vs* 691±38.6 cells to invade (*P*=0.0333 in comparison to TCP). Like 5-HETE and 15(S)-HETE, 12(S)-HETE was only able to release the invasive block induced by EPA, resulting in 935.9±49.6 *vs* 774.8±40.3 cells invading (*P*=0.01944) as compared to EPA control.

Prostaglandin E_2_ was shown to be a potent stimulator of PC-3 invasion, with 10 ng ml^−1^ inducing similar levels to AA (915.42±64.5 *vs* 997.8±55.5; *P*=0.3431). Addition of 10 ng ml^−1^ PGE2 to an invasion blocked system, with 10 *μ*M AA acting as the main stimulus and 20 *μ*M of either EPA, DHA or 8 *μ*M NS-398 blocking invasion (Figure 6D), resulted in complete recovery of AA-induced invasion (997.8±55.47 *vs* 1022.8±84.7 (*P*=0.8042), 911.8±46 (*P*=0.2452), 974.3±14 (*P*=0.6841) for AA *vs* EPA+PGE2, DHA+PGE2 and NS-398+PGE2, respectively).

### Only EPA inhibits invasion towards BMS

**Figure 7:**
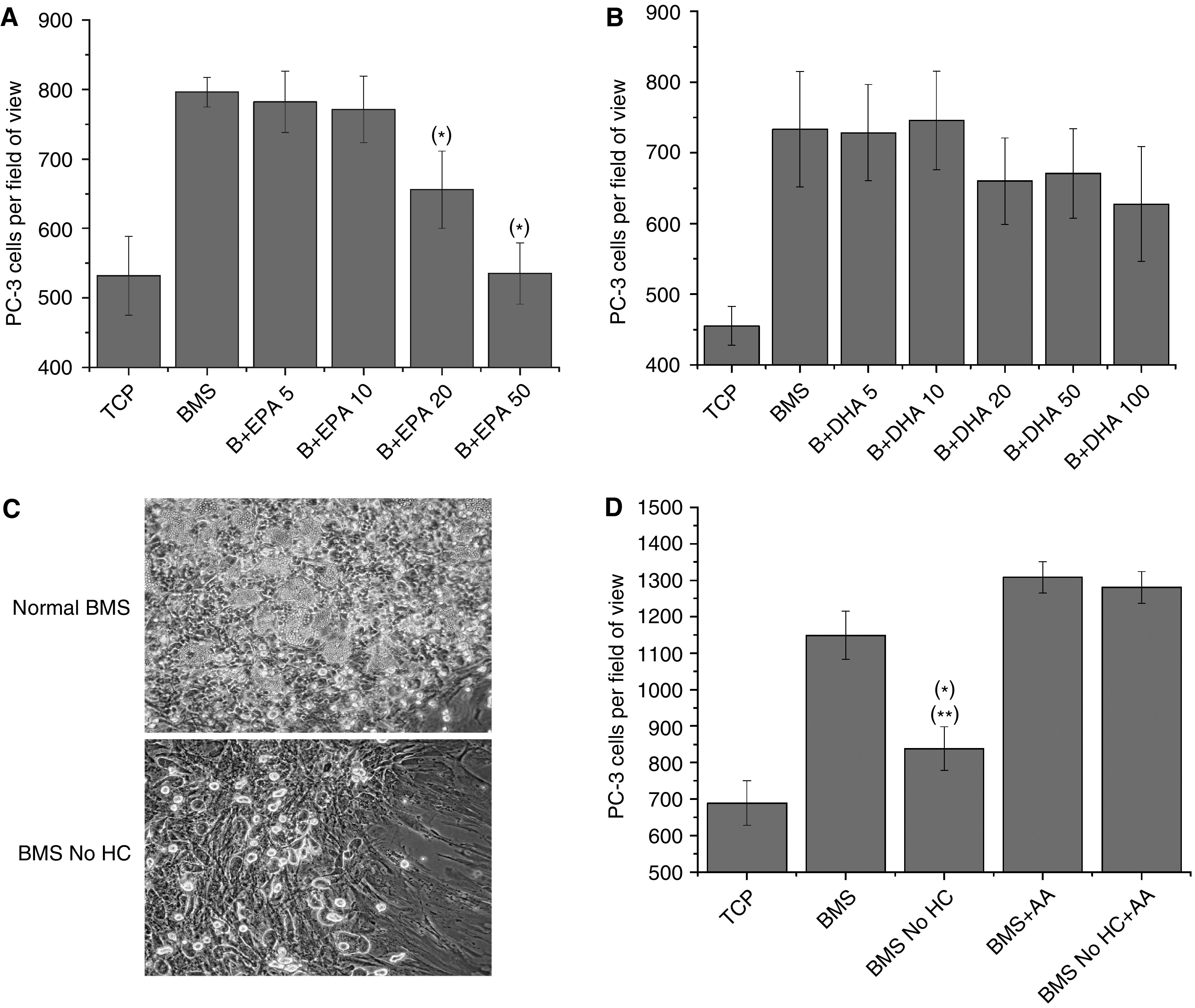
PC-3 cell invasion towards BMS is dependent on bone marrow adipocytes and *ω*-6 metabolism. (**A**) PC-3 cell invasion towards BMS in the presence of escalating doses (5–50 *μ*M) of EPA. (**B**) PC-3 cell invasion towards BMS in the presence of escalating doses (5–100 *μ*M) of DHA. Data represents mean number of cells per field of view plus standard error bars generated from three independent experiments (*n*=3). (**C**) Photomicrographs of a 5-week-old human bone marrow culture, grown in the presence of 0.5 *μ*M hydrocortisone, showing the presence of haemopoietic centres and adipocytes, or in the absence of hydrocortisone, showing stromal development and the absence of adipocytes. (**D**) PC-3 cell invasion towards BMS, grown in the presence or absence of 0.5 *μ*M hydrocortisone (No HC), with or without 10 *μ*M AA. Data represents mean number of cells per field of view plus standard error bars generated from three independent experiments (*n*=3). ^*^=*P* value ⩽0.05 as compared to BMS; ^**^=*P* value ⩽0.05 as compared to TCP.

To determine the potential of *ω*-3 inhibition of malignant PEC invasion towards human BMS, *in vitro* co-culture assays were set up utilising human BMS as the target for invasion in the presence of escalating concentrations of either EPA or DHA (Figure 7A and B). Eicosapentaenoic acid at concentrations ⩾20 *μ*M reduced invasion toward BMS (655.8±55.8 (*P*=0.04085) and 535.3±44 (*P*=0.0003) *vs* 796.2±21.4 at 20 and 50 *μ*M, respectively) as compared to BMS. However, this was not observed with DHA at concentrations up to 100 *μ*M (*P*=0.2905 as compared to BMS).

### AA recovers invasive ability of depleted BMS

To address the potential role of AA in the stimulation of invasion towards BMS *in vivo*, *in vitro* co-culture invasion models were constructed using long-term human BMS grown in the presence or absence of hydrocortisone, essential for the formation of adipocytes in the BMS (Toogood *et al*, 1980) and hence haemopoiesis (Dexter *et al*, 1977). We utilised these cultures as a source of lipid-free BMS. Figure 7C shows photomicrographs of 5-week-old human BMS cultures from the same donor grown in the presence or absence of 0.5 *μ*M hydrocortisone. Both cultures developed similar confluent BMS fibroblasts. However, in the absence of hydrocortisone there were no signs of haemopoiesis or colonies of adipocytes.

The ability of the hydrocortisone-depleted BMS to act as a stimulant for invasion was then assessed in comparison to normal BMS and the ability of AA to recover invasion was determined (Figure 7D). Hydrocortisone-depleted BMS weakly stimulated PC-3 invasion through Matrigel (838.6±59.6 *vs* 688.5±61.1; *P*=0.1096 as compared to TCP) but was a significantly weaker stimulus than control BMS (*P*=0.0059). Addition of 10 *μ*M AA to the hydrocortisone-depleted BMS restored the ability of this BMS to induce invasion, with measured levels of invasion comparable to the level induced by normal BMS also supplemented with 10 *μ*M AA (1280±43 *vs* 1308±42.9; *P*=0.655).

## DISCUSSION

The role of diet, especially PUFAs, in CaP has been a highly controversial subject with epidemiological studies generating conflicting information as to the effect of *ω*-6 and *ω*-3 PUFAs in the pathogenesis and progression of CaP. *In vitro* studies examining the role of both families of PUFAs in CaP have provided some insight into the potential mechanisms of their effect. Metabolism of the essential *ω*-6 PUFA LA leads to the production of AA, metabolites of which have been shown, in the case of 5(S)-HETE, to protect PEC from apoptosis and to induce proliferation, and in the case of 12(S)-HETE to induce endothelial invasion and angiogenesis *in vivo*, leading to the formation of larger tumours. These effects have been shown to be abrogated by the use of *ω*-3 PUFAs (Rose and Connolly, 1999). In this study we have addressed for the first time the role of PUFAs in stimulating BMS metastatic migration, because of the known predilection that prostate cancer has for the haemopoietic bone marrow. This study is the first to demonstrate that *ω*-3 PUFAs have an inhibitory effect on cell migration *in vitro* and thus potentially metastasis.

The means by which cancer cells migrate in a site-specific manner, and in particular, the reasons underlying the predilection that some cancers such as prostate cancer have for the red bone marrow are poorly understood. There are a number of potential factors which may be influential but presently, there is no specific known mechanism. The CXCR4/SDF-1 axis has been postulated as being critical in this process (Taichman *et al*, 2002; Sun *et al*, 2003) but we have previously shown that this forms only a part of the invasive stimulus generated by the BMS (Hart *et al*, 2005). The BMS is a lipid-rich environment, with the presence of adipocytes being of critical importance for the haemopoietic process (Dexter *et al*, 1977). Lipid-related studies of the BMS microenvironment have shown that the lipid component comprises of various fats including oleic acid (35.2±4.9%), palmitic acid (27.8±2.5), palmitoleic (8.1±2.7%) stearic acid (6.3±1.4%) and linoleic acid (12.3±2.7–15.3±2.9%) (Sumida, 1965; Denizot *et al*, 1999). Arachidonic acid, a metabolite of LA, is also present within the BMS although the concentration varies from 2.5±0.9% in the BMS plasma to 9.52±0.4% within the cellular component (Denizot *et al*, 1998). As the body ages the amount of fatty tissue/yellow bone marrow increases (up to 10% every decade (Compston, 2002)), along with a notable increase in adipocytes (Meunier *et al*, 1971; Rozman *et al*, 1989). Using our co-culture *in vitro* models of BMS invasion, we have shown that the bone metastatic CaP cell line PC-3 actively seek out and move towards adipocyte-rich regions of the BMS. In the presence of bone marrow adipocytes the PC-3 cells were observed to take up lipids actively and in a time-dependent manner (Figures 1, 2 and 3). The role of lipid uptake is currently unclear but it is possible that the lipid may be utilised as an energy substrate to meet the needs of the accelerated cellular metabolism, known to be a feature of the metastasising malignant cell, and indeed it has been shown that prostate cancer cells interact with adipocytes. Tokuda *et al* (2003) showed that PC-3/adipocytes co-cultures induced both PC-3 proliferation and differentiation. Conversion of LA to AA leads to the generation of AA metabolites, PGE2, 5-HETE, 12(S)-HETE and 15(S)-HETE. Both PGE2 and 5-HETE have been shown to have CaP proliferative properties. Studies by Hughes-Fulford *et al* (2005) demonstrated that ⩽10 *μ*M AA induced PC-3 proliferation by elevating expression of cPLA2 and COX-2, fivefold and threefold respectively, with subsequent increased levels of PGE2. 12(S)-hydroxyeicosatetraenoic acid also increased prostate tumour growth by increasing angiogenesis with subsequent decrease in necrosis, but it has also been shown to have varied roles in the metastatic processes in CaP, including enhancing cell motility (Honn *et al*, 1994; Gao *et al*, 1995). Unlike PGE2, 5-HETE and 12(S)-HETE, 15(S)-HETE is associated with benign prostate tissue. Formation of 15(S)-HETE is reduced in the majority of CaP (Shappell *et al*, 1999) and it acts as a PPAR*γ* agonist (Shappell *et al*, 2001), an effect which has been shown to inhibit proliferation of breast (Mueller *et al*, 1998), colon (Brockman *et al*, 1998) and bladder (Brockman *et al*, 1998) carcinoma cell lines.

As a major component of the lipid composition of normal BMS adipocytes is LA and its metabolite AA (Sumida, 1965; Denizot *et al*, 1998; Denizot *et al*, 1999) and that the level of AA decreases in the prostate cancer patients (Mamalakis *et al*, 2002), we sought to determine the rate of lipid uptake. PC-3 cells cultured in the presence of 10 *μ*M AA and stained with Nile Red rapidly took up the PUFA from the local environment (within 15 min), reaching a maximum level of uptake after 45 min. The level of AA then diminished over time suggesting metabolism of the AA into PGE2 (Hughes-Fulford *et al*, 2005) or HETEs. This potential rapid metabolism of AA, correlating with patient data (Mamalakis *et al*, 2002; Faas *et al*, 2003), suggests that AA cyclooxgenase and lipoxygenase products are potentially important components of the cancer process.

The study by (Mamalakis *et al*, 2002) also showed that despite a reduction of AA in prostatic tissues between CaP and BPH patients there was no significant difference in the levels of AA within the adipose tissue. We therefore hypothesised that the level of AA within the adipocyte-rich BMS may be maintained in CaP patients and act as an attractant for metastatic PEC. Using our in house *in vitro* invasion assays we showed that AA was a potent stimulator of invasion, inducing similar levels of invasion as to those seen using BMS. This series of experiments showed that AA is a far stronger stimulator of invasion than SDF-1 (Hart *et al*, 2005) and that this is possibly the predominant signal for invasion of the BMS, leading to the characteristic disturbance of the skeletal and bone marrow metabolism.

The importance of the AA signal from the BMS was further confirmed in our *in vitro* co-culture models utilising primary human BMS cultures grown in the presence or absence of hydrocortisone (Figure 7C and D). Toogood *et al* (1980) showed that mesenchymal progenitors within the BMS cultured in the absence of hydrocortisone do not differentiate into adipocytes. In the absence of adipocytes, BMS induced PC-3 invasion but at a significantly lower level than adipocyte-rich BMS. The small amount of invasion observed was possibly due to the production of other factors such as SDF-1, which has previously been shown to induce PC-3 invasion, by the BMS (Hart *et al*, 2005). Addition of 10 *μ*M AA completely restored the ability of adipocyte-free BMS to induce PC-3 invasion demonstrating the potency of AA to induce invasion.

Omega-3 PUFAs, especially the marine PUFAs, EPA and DHA, have been shown to inhibit the proliferation of both prostate and breast cancer cells *in vitro* and *in vivo*. The mode of action of *ω*-3 PUFAs is by competing with AA for the cyclooxygenases and the lipoxygenases, leading to the production of metabolically inactive products such as *Δ*^17^-6-keto-PGF and TXB_3_ (reviewed in Rose and Connolly, 1999). Furthermore, dietary intake of *ω*-3 PUFAs has been associated with a decreased risk in developing aggressive/metastatic CaP (Terry *et al*, 2001; Augustsson *et al*, 2003). Epidemiological evidence now points to the importance of the *ω*-3 : *ω*-6 ratio within the diet and a decrease in this ratio is associated with increased risk of aggressive disease (Rose and Connolly, 1999). This is most noticeable in the diets of Japanese and Eskimos who traditionally had a high *ω*-3 : *ω*-6 ratio due to high fish intake. This has changed over the last couple of decades to a more Western low *ω*-3 : *ω*-6 diet with a subsequent increase in the risk of both breast (Wynder *et al*, 1991) and prostate cancer (Lanier *et al*, 1976; Lanier *et al*, 1996).

Here we demonstrate that the *ω*-3 PUFAs DHA and EPA are strong inhibitors of invasion towards AA capable of blocking invasion at a ratio of 1 : 2 *ω*-3 : *ω*-6 (Figure 5). The predominant block is in the production of PGE2 by COX-2 as the addition of 10 ng ml^−1^ PGE2 to an *ω*-3 or NS-398 COX-2 inhibitor blocked system restores the level of PC-3 invasion (Figure 6D). Therapeutic blockade of PGE2 production using COX inhibitors such as NSAIDs or specific COX-2 inhibitors such as celecoxib, rofecoxib and NS-398 have shown potential to inhibit both tumour growth and metastasis in experimental animal models (Dandekar and Lokeshwar, 2004; Patel *et al*, 2005). However, there are concerns after observations that COX-2 inhibitors increase the risk of cardiovascular events during both the VIGOR and APC trials (reviewed in Krotz *et al*, 2005; Luo *et al*, 2005). Unlike the synthetic COX-2 inhibitors, EPA and DHA are associated with a reduced risk of cardiovascular disease and sudden cardiac death (Holub and Holub, 2004; Harrison and Abhyankar, 2005). Trials have shown that the early administration of 1 g day^−1^ *ω*-3 PUFA supplements reduced the risk of sudden cardiac death by 45% and the overall mortality by 20% (Marchioli *et al*, 2002) and that doses of up to 4 g day^−1^ doses can be tolerated (reviewed in Harrison and Abhyankar, 2005). Therefore we propose that there is particular scope for the use of dietary DHA and EPA not only to effect proliferation but also in the anti-metastatic treatment of CaP.

The data suggests that both DHA and EPA may also act on different pathways involved in invasion. Figure 5 shows that both 5 *μ*M DHA and EPA reduced PC-3 invasion to levels similar to that of nondirectedmigration but the addition of the lipoxygenase products 5, 12(S) and 15(S)-HETE show different patterns of recovery (Figure 6). Addition of HETEs was able to partially restore the invasive effect of AA in the presence of EPA but did not remove the DHA block. Surprisingly 15(S)-HETE, which is not produced from AA by PC-3 owing to loss of 15-LOX-2 expression and plays a role in the suppression of *ω*-6-induced PEC proliferation (Shappell *et al*, 1999; Shappell *et al*, 2001), partially restored EPA-blocked PC-3 invasion. However, addition of PGE2 to systems blocked by either EPA or DHA completely restored invasion towards either AA or BMS. This difference was highlighted by the potential of *ω*-3 PUFAs to inhibit PC-3 invasion towards primary BMS cultures (Figure 7A and B). Only EPA was able to block invasion towards BMS although only at higher concentrations (20–50 *μ*M) than observed in the monoculture invasion assays. This may be due to the presence of PGE2 and HETEs already within the co-culture which is due to the metabolism of *ω*-6 PUFAs by cells within the BMS. Within this system, however, DHA was unable to block the BMS invasive stimuli, even at a final concentration of 100 *μ*M.

In summary we have provided *in vitro* evidence supporting the epidemiological data that the dietary ratio of *ω*-3 : *ω*-6 is crucial in determining the risk of metastatic disease in CaP. Arachidonic acid is a potent stimulator of PEC invasion and is a major component of the stimulus directing metastatic PEC to the BMS to form bone metastases. As the epidemiological data suggests, increasing the ratio of *ω*-3 : *ω*-6 PUFAs, in particular increasing the amount of EPA in the diet, can inhibit the metastatic process by blocking the production of PGE2 and therefore reducing the risk of aggressive disease.
