# Supplementary figures and images for: Promotion of prostatic metastatic migration towards human bone marrow stoma by Omega 6 and its inhibition by Omega 3 PUFAs
Source: Br J Cancer. 2006 Mar 7;94(6):842–53. doi: 10.1038/sj.bjc.6603030 (PMC2361380; doi:10.1038/sj.bjc.6603030)

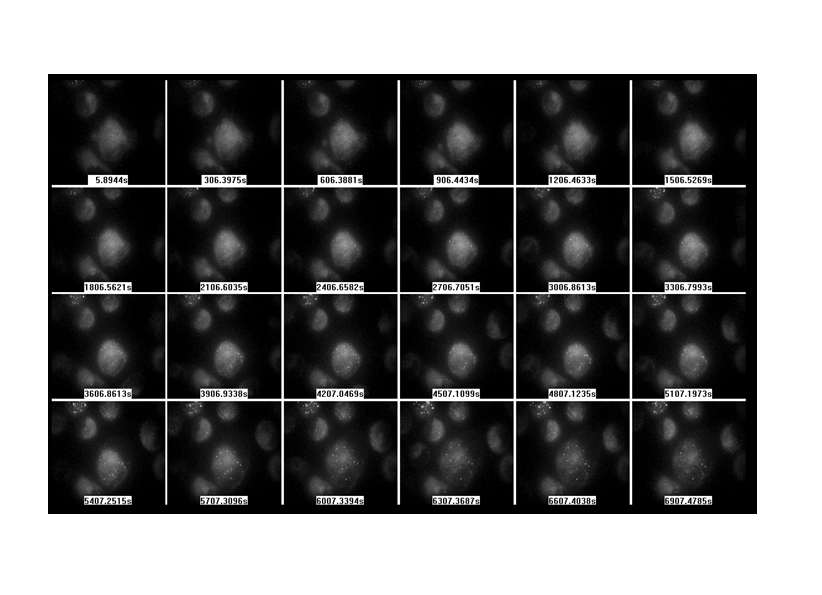

Supplement: Supplementary Figure 1b [file 94-6603030x2.tif]

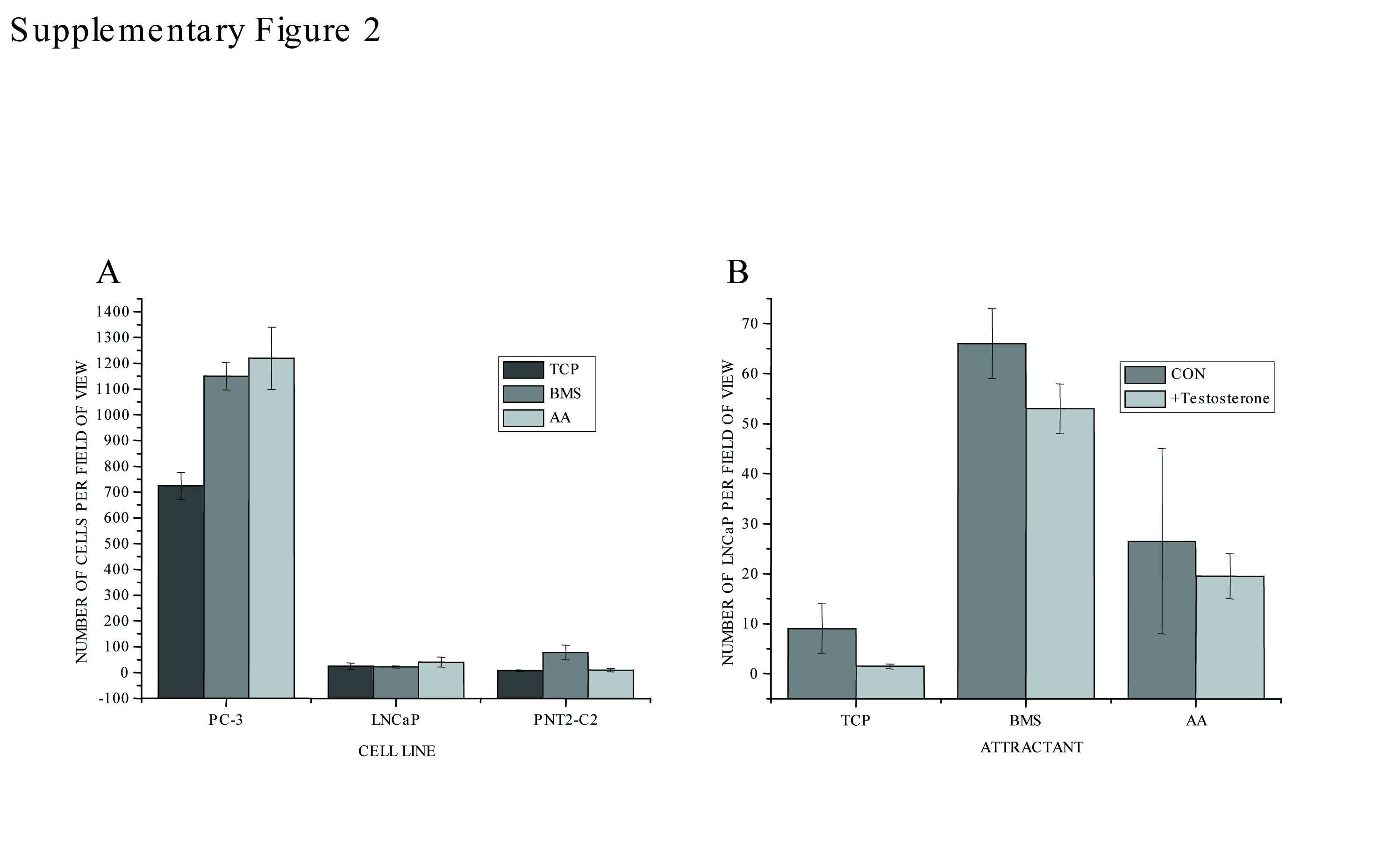

Supplement: Supplementary Figure 2 [file 94-6603030x3.tif]
