# Supplementary material for: Promotion of prostatic metastatic migration towards human bone marrow stoma by Omega 6 and its inhibition by Omega 3 PUFAs
Source: Br J Cancer. 2006 Mar 7;94(6):842–53. doi: 10.1038/sj.bjc.6603030 (PMC2361380; doi:10.1038/sj.bjc.6603030)
Supplement: Supplementary Figure Legends [file 94-6603030x4.doc]

**Supplementary Figure Legends**

Figure 1. PC-3 cells take up Arachidonic without the aid of albumin carrier from the surrounding environment. AA uptake by PC-3 cells cultured in the presence of 0.5% Nile Red followed over time by a PerkinElmer Ultraview situated on a Zeiss Axiovert 200M with a full environmental chamber at 37°C at 630x magnification. Images were captured every 5 minutes following addition of 10 µM AA at 306 seconds. (A) Movie compilation of each 5 minute interval. Post addition of AA, brightly fluorescent lipid droplets form which is accompanied by a change in PC-3 morphology, from mesenchymal to amoeboid.

(B) Photomicrographs following the uptake of lipid by a PC-3 showing each individual 5 minute timeframe from the movie in Figure 1A. Maximal staining of the cells is observed between 45 and 50 minutes after the addition of AA. 60 minutes thereafter, the overall fluorescence of the cell decreases and the AA appears to accumulate in Nile Red staining droplets within the cytoplasm, particularly in areas surrounding the nucleus. These droplets are maintained over 115 minutes in the culture although the overall staining intensity appears to diminish over time.

Figure 2. Arachidonic acid does not induce PNT2-C2 or the androgen sensitive cell line LNCaP to invade. Matrigel coated cell culture inserts were placed in a 24 well plate containing 1ml of DMEM/0.1% fatty acid free BSA with either plain tissue culture plastic (TCP negative control), BMS (positive control) or 10μM of AA in the presence or absence of 2x10^-4^ μM dihydrotestosterone. Serum starved PC-3, LNCaP or PNT2-C2 cells were seeded at 2x10^5^ per insert then incubated at 37°C for 18h after which inserts were fixed and stained in 2% crystal violet and counted according to manufacturer’s instructions using a grid graticule. (A) Histogram showing the invasive stimulatory effect of 10μM AA on the malignant prostate cell lines PC-3 and LNCaP and the benign prostate cell line PNT2-C2. Data represents mean number of cells per field of view plus standard error bars generated from 2 independent experiments (n=2). (B) Histogram showing the invasive stimulatory effect of 10μM AA in the presence of 2x10^-4^ μM dihydrotestoerone on LNCaP cells. Data represents mean number of cells per field of view plus standard error bars generated from 2 independent experiments (n=2).
